# A neutral model for the loss of recombination on sex chromosomes

**DOI:** 10.1098/rstb.2020.0096

**Published:** 2021-08-30

**Authors:** Daniel L. Jeffries, Jörn F. Gerchen, Mathias Scharmann, John R. Pannell

**Affiliations:** Department of Ecology and Evolution, University of Lausanne, 1015 Lausanne, Switzerland

**Keywords:** sex chromosomes, recombination, neutral model, sexually antagonistic selection

## Abstract

The loss of recombination between sex chromosomes has occurred repeatedly throughout nature, with important implications for their subsequent evolution. Explanations for this remarkable convergence have generally invoked only adaptive processes (e.g. sexually antagonistic selection); however, there is still little evidence for these hypotheses. Here we propose a model in which recombination on sex chromosomes is lost due to the neutral accumulation of sequence divergence adjacent to (and thus, in linkage disequilibrium with) the sex determiner. Importantly, we include in our model the fact that sequence divergence, in any form, reduces the probability of recombination between any two sequences. Using simulations, we show that, under certain conditions, a region of suppressed recombination arises and expands outwards from the sex-determining locus, under purely neutral processes. Further, we show that the rate and pattern of recombination loss are sensitive to the pre-existing recombination landscape of the genome and to sex differences in recombination rates, with patterns consistent with evolutionary strata emerging under some conditions. We discuss the applicability of these results to natural systems.

This article is part of the theme issue ‘Challenging the paradigm in sex chromosome evolution: empirical and theoretical insights with a focus on vertebrates (Part I)’.

## 1. Introduction

A strikingly convergent pattern among species with genetic sex determination is the loss of recombination between their sex chromosomes [1]. This results in a lower efficacy of purifying or positive selection and opens the door to the accumulation of deleterious mutations, loss of function and altered patterns of gene expression. Given these important implications, the frequent loss of recombination on sex chromosomes across such a diverse range of taxa [2] demands an explanation. What is it about the regions adjacent to sex-determining (SD) genes that predisposes them to the loss of recombination? Despite the considerable attention this question has received, we still lack a fully satisfying answer.

So far, several hypotheses have been put forward, as recently reviewed in Ponnikas *et al*. [3]. Charlesworth & Wall [4] proposed that selection could favour expansion of the non-recombining region (NRR) if it encompasses deleterious recessive mutations, as they would then be sheltered by the fixed heterozygosity of the sex-linked region. More recently, Úbeda *et al*. [5] proposed that recombination suppression could be selected for in cases where the SD locus arises near to a meiotic driver or a drive suppressor, in order to ensure balanced sex ratios. Finally, the most widely cited explanation for suppressed recombination on sex chromosomes is sexually antagonistic (SA) selection. Under this hypothesis, selection should favour the suppression of recombination between the SD locus and a physically linked SA locus (i.e. a locus with opposite fitness effects in the two sexes). This is because increasing linkage disequilibrium (LD) between these loci will maintain each allele at the SA locus in its appropriate genetic background, thus resolving intragenomic conflict. First presented by Fisher [6], this theory was later expanded theoretically by Charlesworth & Charlesworth [7], Bull [8] and Rice [9]. The SA selection hypothesis is appealing because it makes immediate intuitive sense for species in which males and females differ in morphology, behaviour, life history or physiology.

Despite the plausibility of the SA hypothesis, we still lack strong evidence for a causal relationship between selection of any kind and the loss of recombination on sex chromosomes. In several taxa, it seems that regions of low recombination likely pre-dated (and perhaps facilitated) the linkage of SA variants, meiotic drivers or deleterious recessive alleles to a SD locus [10–15]. Furthermore, recombination loss is seen in systems where sex chromosome turnovers are extremely common, for example in several families of fish [16–18]. Selection for linkage between a SD locus and, for instance, a SA locus that confers an advantage to the heterogametic sex, is expected to have a stabilizing effect on that SD system, as any new SD system that arises would have to overcome that advantage [19,20]. Thus, while it seems likely that selection has played a role in the evolution of suppressed recombination around SD loci, it remains unclear how important these processes have been.

Each of the hypotheses summarized above supposes an adaptive reason for the loss of recombination on sex chromosomes and they all invoke selection for genetic linkage with the sex determiner as a mechanism. By contrast, the possibility that recombination has been lost on sex chromosomes for non-adaptive reasons has received little attention. While it has been discussed briefly by Charlesworth [1,21] and Kent *et al.* [22], non-adaptive explanations have only been addressed at any length in two studies. Bengtsson & Goodfellow [23] developed a theoretical model (later coined the ‘attrition’ model [24]) to assess how neutral diversity can be expected to behave in the pseudoautosomal region (PAR) of mammalian sex chromosomes. They found that sequence divergence between the sex chromosomes should accumulate in the absence of selection if recombination rate was of the same order of magnitude as the mutation rate or lower.

More recently, Ironside [25] proposed a verbal model concerning the role of neutral inversions. On an autosome, a neutral inversion will momentarily prevent recombination in heterozygous individuals but will ultimately be fixed or lost via genetic drift. In either case, recombination will be free to resume. By contrast, if such an inversion arises and fixes on a Y or W chromosome and either encompasses the SD locus or overlaps with an existing NRR, it will be forever maintained in a heterozygous state by the extreme balancing selection on the SD locus. As such, recombination will be permanently halted in this region. While this model is intuitive, it does not explain the loss of recombination on sex chromosomes where no inversion is observed. But the idea that heterozygosity should be maintained around the SD locus should apply to any mutation that is in strong LD with the SD locus, not just to inversions. Thus, the key concept of Ironside's model could apply much more generally.

**Figure 1.**
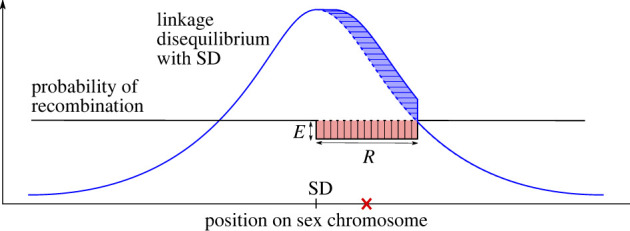
Schematic representation of our model for loss of recombination on sex chromosomes. For a given heterozygous position (red cross), initial recombination probability (*r_*0*i_*, dashed black line) is reduced by a factor of *E* in a window around it of width *W* (red vertical line-shaded area). This in turn increases LD with the SD for a portion of the chromosome (blue curved line and horizontal line-shaded area) and so facilitates the further accumulation of differences between the sex chromosomes. (Online version in colour.)

Here, we propose a model for the non-adaptive loss of recombination on sex chromosomes that encompasses aspects of the models from both Bengtson & Goodfellow [23] and Ironside [25]. In our model, frequency-dependent selection and the genetics of sex determination maintain polymorphism indefinitely at the SD locus itself. If neutral or nearly neutral mutations (henceforth referred to simply as neutral) occur at a rate that is sufficiently high relative to the local recombination rate, then short-range LD should allow the two haplotypes close to the SD locus to begin to diverge [23], as would be the case for any two populations of partially isolated sequences [26,27]. The novelty of our model comes from the notion that heterozygosity at any site reduces the likelihood of recombination at and close to that site. As such, neutral divergence that builds up in LD with the SD locus will, itself, reduce the recombination rate in that region, creating a positive feedback loop and facilitating the build-up of more divergence (figure 1). We suggest that this recursive process might eventually lead to a point where recombination no longer occurs at an appreciable level in the regions adjacent to the SD locus.

**Figure 2.**
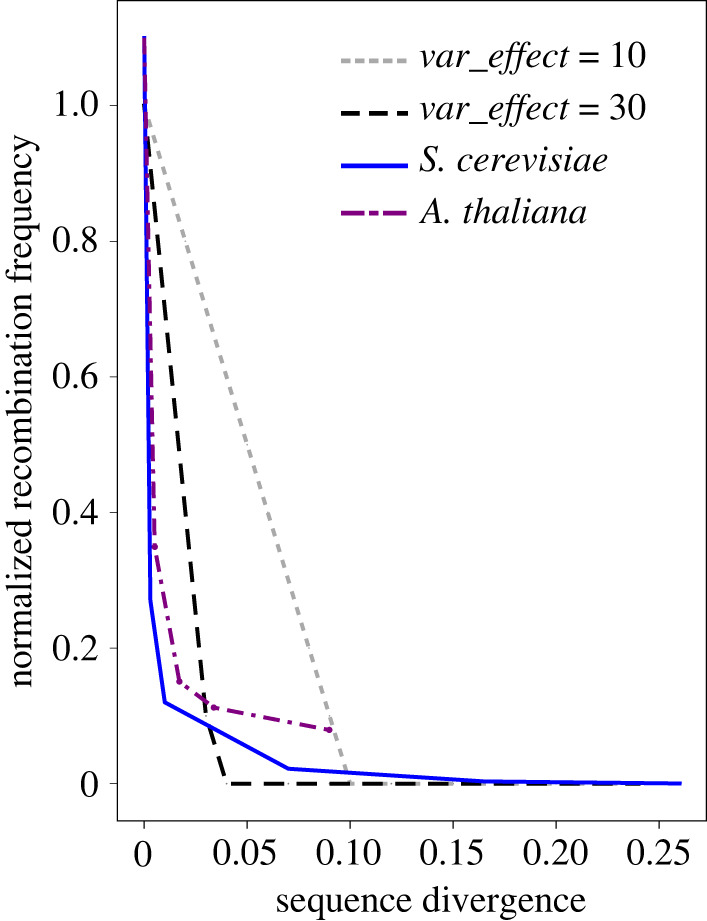
The relationship between sequence divergence and normalized recombination rate found in *Saccaromyces cerevisiae* (blue line; [31]) and *Arabidopsis thaliana* [33]. The light and dark grey lines represent the relationship between sequence divergence and recombination probability as specified in our simulations using the *E* parameter. (Online version in colour.)

The negative relationship between recombination rate and sequence divergence has a strong empirical basis and is a widespread characteristic of genome evolution. Recombination between homologous sequences is fundamental to the reproductive process across much of the tree of life, but it opens the way to ectopic recombination (between non-allelic sites), which can result in massive, often lethal structural rearrangements and aneuploidy. It is thus not surprising that the recombination machinery has evolved to avoid recombination between dissimilar sequences, specifically via strict homology searching [28] and the mismatch repair pathway, which is conserved across prokaryotes and eukaryotes [29]. While the exact biomolecular processes involved are complex and differ between organisms, one apparent consistency is the significant inhibitory effect that even a few mismatched bases can have on recombination rates between homologous sequences [30–33] (figure 2). Given the maintenance of constant heterozygosity at SD loci, this could play a significant role in the evolution of sex chromosomes, yet to our knowledge, is still to be explored in this context.

Here, we used computer simulations to identify conditions under which a NRR on a Y-chromosome could arise and expand, and assess the applicability of this model to the natural world. Intuitively, divergence between sex chromosomes should accumulate adjacent to the SD locus as long as the mutation rate is sufficiently high relative to the recombination rate, and the rate at which neutral divergence builds up should be slowed with increasing population sizes. We therefore focused especially on these three core parameters. We also performed simulations to test the effects of both heterochiasmy (a difference in the rate and distribution of recombination events between sexes) and male-biased mutation rates, as both are common in eukaryotes, and are expected to influence sex chromosome evolution [10,12,34,35]. Lastly, as recombination also varies substantially over the genome, we examine the effect of a non-uniform recombination landscape, including local hotspots and coldspots (shared by both sexes), on the rate and pattern of recombination loss.

## 2. Simulation methods

To test our model, we simulated a 100 kb long stretch of DNA in a dioecious population with a fixed number of randomly mating diploid individuals and non-overlapping generations. We assumed an XY system of sex determination, with sex determined by a single base mutation located midway along with the simulated DNA sequence. In male meioses, half of the gametes received a Y male-determining allele, while the other half received its X homologue. Simulations began with no polymorphism in the population other than the SD nucleotide. In each generation, neutral point mutations were allocated at random positions along the sequence at rate *μ*. If a mutation fell at an already polymorphic site, that mutation was reallocated. We also performed a test set of simulations in which we simply discarded mutations that landed at already polymorphic sites and found close agreement between the two approaches (see electronic supplementary material, figure S1).

Recombination events occurred at a sequence-wide rate of *r*, with their locations along the sequence, *i*, being drawn from a vector of recombination probabilities at each position *r_i_*. Each simulation started with the vector *r_i_* set with the initial (baseline) recombination probabilities at generation zero, *r*_0*i*_. Based on this initial vector, recombination probabilities were calculated for each meiosis as a function of divergence between haplotypes at each position. Specifically, for a given target site *i*:

$$r_{i}=r_{0i}\times \left(1-\frac{Eh}{W}\right),$$

where *E* is the factor by which each heterozygous position reduces recombination probability, *h* is the number of heterozygous positions in the given window and *W* is the width of the window centred on position *i*.

We assumed a linear decline in the recombination rate with sequence divergence (figure 2). After exploring the sensitivity of our model to parameter values, we set *W* = 1000 and *E* = 30, with a single mutation thus reducing the baseline recombination rate by 3% (*E = 30*) for a 1000 bp window around it. To explore the impact of male mutation bias (MMB), we ran simulations in which male versus female rates differed by a constant factor for a given run, while maintaining a constant sex-averaged mutation rate at *μ* = 7.5 × 10^−6^. We explored the effect of the initial recombination landscape (*r_i_*) by comparing three scenarios that differed in the spatial pattern of the initial recombination probabilities: uniform, heterochiasmatic and hotspot/coldspot. In the heterochiasmatic model, the male recombination rate was higher towards the ends of the simulated sequence and lower at the centre, while in the hotspot/coldspot model, initial recombination probabilities fluctuated in a sinusoidal manner along the sequence in both sexes. In all cases, global recombination rates (averaged across the whole sequence and across both sexes) remained constant.

Because of constraints on computer time, we were limited to simulating small populations only (*N* = 50–200); in the Results and discussion (§3), we consider the limitations this poses for our interpretations. Given the low population sizes investigated, we varied the mutation rate *μ* and initial sequence-wide recombination rate *r* around *N*-scaled rates (*θ* = 4 *Nμ* and *ρ* = 4 *Nr*) that correspond to values typical for eukaryotes [36,37]. In simulations exploring different *N*, we kept *θ* and *ρ* constant by scaling *μ* and *r* accordingly. Simulations were run for 10 000, 20 000 or 30 000 generations depending on the parameters or scenarios being tested. All parameter combinations and simulation details are set out in table 1.

**Table 1.** Details of parameter values used in all simulations performed. Each row summarizes all parameter ranges used for each set of simulations. The focal test parameter of each set is highlighted in italics. Theoretical linkage map lengths for each simulation are given below the recombination rate.

simulation set	*N*	*generations (x1000)*	*μ*	*r*	*θ*	*ρ*	*W*	*E*	MMB	ancestral recombination landscape
1	100	*20–30*	*1 × 10^−6^–1 × 10^−5^*	*1 × 10^−7^ –1 × 10^−6^* (1–10 cM)	4 × 10^−4^–4 × 10^−3^	4 × 10^−5^–4 × 10^−4^	1000	10 & 30	1	uniform
2	*50–200*	20	3.75 × 10^−6^–1.5 × 10^−5^	5 × 10^−8^–2 × 10^−7^ (0.5–2 cM)	3 × 10^−3^	4 × 10^−5^	1000	10	1	uniform
3	100	20	7.5 × 10^−6^	1 × 10^−7^ (1 cM)	3 × 10^−3^	4 × 10^−5^	1000	*6–14*	1	uniform
4	100	20	7.5 × 10^−6^	1 × 10^−7^ (1 cM)	3 × 10^−3^	4 × 10^−5^	*100–1000*	10	1	uniform
5	100	10	7.5 × 10^−6^	1 × 10^−7^ (1 cM)	3 × 10^−3^	4 × 10^−5^	1000	10	1	*heterochiasmatic*
6	100	10	7.5 × 10^−6^	1 × 10^−7^ (1 cM)	3 × 10^−3^	4 × 10^−5^	1000	10	1	*hotspot / coldspot*
7	100	20	7.5 × 10^−6^	1 × 10^−7^ (1 cM)	3 × 10^−3^	4 × 10^−5^	1000	10	*1–10*	uniform

For each set of simulations, we recorded the size of the NRR every 100 generations. This was defined as a contiguous region containing the SD in which recombination probabilities in the vector *r_i_* had reached zero in the entire population. We simultaneously recorded average sequence divergence in female meioses (i.e. between X–X pairs; dXX), the average sequence divergence in male meioses (i.e. between X-Y pairs; dXY) and the male–female *F*_ST_ at each position on the simulated stretch of DNA. All reported statistics (dXX, dXY, *F*_ST_, recombination probabilities and NRR size) were averaged over 25 replicate runs.

Simulations were implemented in C++11. Full details and instructions for the installation and running of the model can be found on GitHub (https://github.com/mscharmann/sexrecevo).

## 3. Results and discussion

### (a) Key results of the simulations

**Figure 3.**
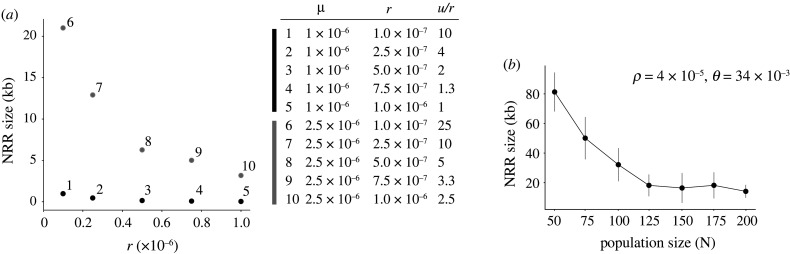
(*a*) Parameter space exploration for *μ* and *r* when *N* = 100, *E =* 30 and *W* = 1000. Each point represents the averaged NRR size across all 25 iterations for each simulation after 20 000 generations. (*b*) Effect of population size on the size of the NRR after 20 000 generations. Plotted points and vertical lines represent the mean and standard deviation of the size of the NRR across all 25 iterations of each simulation.

Our simulations identified conditions that promoted the evolution of reduced recombination around the SD locus, and those in which reduced recombination did not evolve. As predicted by the analytical work of Bengtsson & Goodfellow [23], recombination was lost more rapidly when *μ* was high relative to *r* (e.g. *N* = 100, *μ* ≥ 1 × 10^−06^, *r* ≤ 1 × 10^−06^, figure 3*a*). Importantly, however, the rate at which recombination decreased depended not just on the relative values of *μ* and *r*, but on their absolute values. This is best seen by comparing simulations 1 and 7 in figure 3*a*. In both, *μ/r* = 10, but mutation and recombination rates are 2.5-fold higher in simulation 7. These conditions resulted in a faster initiation of recombination loss and a faster subsequent rate of loss in simulation 7 than in simulation 1 (electronic supplementary material, figure S2a). Given the above, it is somewhat surprising that simulations assuming a greater mutation rate in males than females revealed no significant increase in the rate at which the NRR expanded, with variation between the 25 simulations for each value of MMB being far greater than between sets of simulations with differing MMB values (electronic supplementary material, figure S2e). However, we note that, while the Y chromosome might experience an elevated mutation rate relative to the X chromosome, the X spends only a third of its time in males, so should experience the lowest mutation rate of any genome compartment. As recombination loss depends on polymorphism arising on both the Y and the X, it is possible that this reduction in the X mutation rate decreases the overall effect of a male-biased mutation in our model.

Recombination loss was also heavily dependent on population size, taking longer to begin, and progressing more slowly in larger populations (figure 3*b*; electronic supplementary material, figure S1b), presumably because of the slower coalescence for large *N* and thus slower divergence in sequence between homologous segments along with the simulated sequence. Interestingly, the size reached by the NRR over the course of our simulations dropped rapidly with increasing population size but levelled off with population sizes above 125 (figure 3*b*). This likely reflects the relationship between population size and the probability of fixation of neutral mutations [38], although more detailed simulations in which individual allele frequencies are tracked would be required to formally test this hypothesis.

The dynamics of recombination decline under our model are evidently complex and depend on the interaction between *μ, r_i_* and *N*. To appreciate this complexity, consider the fate of a new mutation on one of the gametologs. Once a mutation arises near to the SD locus, it might be lost from the gametolog on which it arose, or it may be fixed there. Alternatively, if recombination between the new mutation and the SD locus transfers it to the alternative gametolog, it may now be fixed or lost from either or both of them. The proportion of new mutations that succumb to each fate should depend on both the local recombination rate and the local effective population size. When both are low, mutations have a good chance of drifting to fixation on one gametolog before recombination communicates the mutation to the other. However, the number of mutations that eventually fix between gametologs is ultimately limited to a proportion of all mutations that arise, thus, the rate at which recombination is lost will always be inherently linked to the mutation rate. In addition, though many mutations will fail to fix between the gametologs, any polymorphism that exists in the intermediary stages of loss or fixation will contribute to heterozygosity and thus potentially further contribute to the reduction of recombination.

### (b) Limitations of the model and simulations

At the heart of our model is the relationship between recombination rate and sequence divergence. We assumed that recombination probability in a given region reduced linearly by a factor of *E* as sequence divergence increased, eventually reaching zero when sequence divergence = 1/*E*. As expected, increasing the effect of each mutation on the reduction of recombination probability (*E*) resulted in recombination loss at a lower value of *μ* for a given *r* (electronic supplementary material, figure S2c). Similarly, increasing the width of the window affected by each mutation (*W*) also resulted in an increase in the rate of recombination loss, though this effect was moderate compared to that of *E* (electronic supplementary material, figure S2d). Outside of these tests, simulations were performed with *E* = 30, which is similar to the initial drop in recombination for low levels of sequence divergence observed in *Saccharomyces cerevisiae* [31], *Arabidopsis thaliana* [33] (figure 2) and mammals [39]. However, in reality, this relationship appears to be more L-shaped, with very rare recombination events occasionally seen at high-sequence divergences (greater than 10%). This L-shaped relationship is likely governed by the molecular mechanisms of heteroduplex formation and the reliance on stretches of 100% sequence identity over a minimal length of DNA (termed ‘minimal efficient processing segment’, MEPS) [40,41]. However, MEPS length is known to differ substantially among taxa, from tens of bases in *S. cerevisiae* [30,32,42,43], to hundreds or thousands of bases in mammals [39,44]. In turn, the relationship between recombination rate and sequence divergence is also likely to be highly variable throughout nature. Our simplified model likely gives us a first-order sense of what might happen given this diverse range of contexts, but it is important to note that rare recombination events at high-sequence divergences could homogenize sex chromosomes [45], reducing the expansion of non-recombination under our model. Therefore, a more nuanced approach to this aspect of our model, for instance using the random walk approach of Fujitani and Kobayashi [46] would undoubtedly be worthwhile in future work.

Our mutational model was also highly simplified compared to natural situations. First, we considered only the effect of heterozygous point mutations on recombination rates and not analogous effects that might arise from a structural variation such as inversions, insertions and deletions, which are likely to suppress recombination more strongly. Second, our simulations did not account for the effect of mutations that, by their nature, inhibit recombination (i.e. regardless of whether or not they are heterozygous). In mammals, for example, recombination initiation and the presence of hotspots are heavily reliant on specific sequence motifs that bind the chromatin-modifying protein PRDM9 [47]. Such motifs number in the tens of thousands in mammalian genomes [48], and so are likely to fall close to a sex determiner wherever one arises. A single mutation at a PRDM9-binding site should substantially reduce the rate of recombination, regardless of whether it is heterozygous or homozygous. It has also been proposed that the accumulation of transposable elements in sex-linked regions could lead to silencing via methylation and chromatin alterations, which would also suppress recombination, regardless of heterozygosity [22]. These additional forms of mutation would surely increase the potential for neutral loss of recombination on sex chromosomes on top of that which we have modelled. As such, our model likely provides a conservative estimate of the parameter space in which we might expect this process to occur at the population sizes simulated.

### (c) Relevance of our model: how often might it apply in nature?

Due to computational limitations, we were only able to consider small populations from 50 to 200 individuals. As shown by our results, and as expected on the basis of the longer coalescent times, neutral loss of recombination occurred more slowly for populations with larger effective sizes. Thus, it is difficult to extrapolate how low the recombination rate and how high the mutation rate would have to be to allow for the neutral expansion of an NRR in more realistic scenarios. We note, however, that while genome-wide recombination rates, mutation rates and effective population sizes vary strongly across eukaryotes, a general trend (from the limited estimates currently available) seems to be that taxa with low population sizes tend to also possess higher mutation rates (figure 5*a*) and lower recombination rates (figure 5*b*).

**Figure 4.**
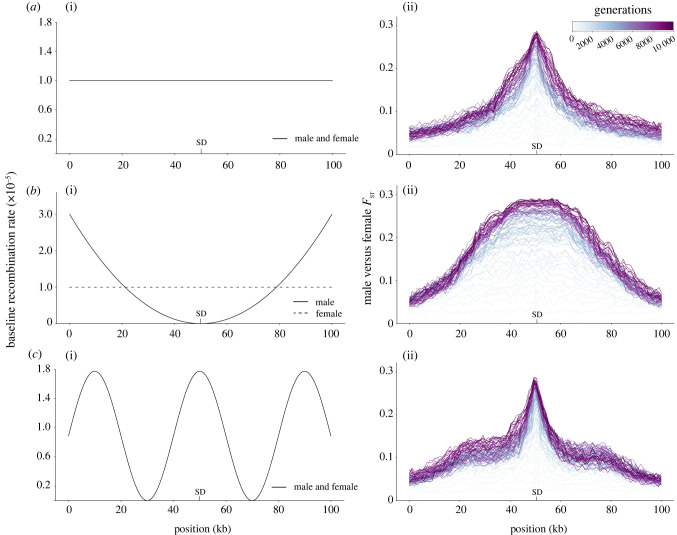
The effect of three different starting recombination landscapes (i) in the rate and pattern of neutral sex chromosome evolution (*F*_ST_; ii). (*a***)** Uniform, (*b***)** heterochiasmatic and (*c***)** hotspot/coldspot starting recombination landscapes were tested. All simulations were run for 10 000 generations, and male versus female *F*_ST_ was averaged over 25 replicates. (Online version in colour.)

More important than genome-wide averages in mutation and recombination rates are local conditions close to the SD. For example, our simulations of a fluctuating recombination landscape showed that recombination loss proceeded quickly through pre-existing coldspots but slowly through hotspots (figure 4*c*). Interestingly, this generated patterns of divergence consistent with evolutionary strata (i.e. large regions across which recombination was lost at roughly the same time [49]). Furthermore, in situations where recombination rates differ between the sexes (either genome-wide or locally), regions in high LD with an SD locus or an existing NRR will experience similarly sex-biased conditions. Again, this was reflected in the results of our simulations of heterochiasmatic recombination rates (figure 4*b*), in which NRR expansion occurred more rapidly compared to simulations with a uniform recombination landscape in both sexes. Lastly, regions that experience LD with the SD will, to a proportional extent, have lower effective population sizes by virtue of their census numbers in the population, with fully sex-linked regions of the X and Y having ¾ and ¼ the effective size of autosomes, respectively. These effective size ratios may be further decreased by factors unrelated to census size: for example, sexual selection [50,51] on the heterogametic sex or mating strategies that increase variance in reproductive success (e.g. polygyny [52]) should tend to reduce the effective size of regions in LD with the SD locus.

**Figure 5.**
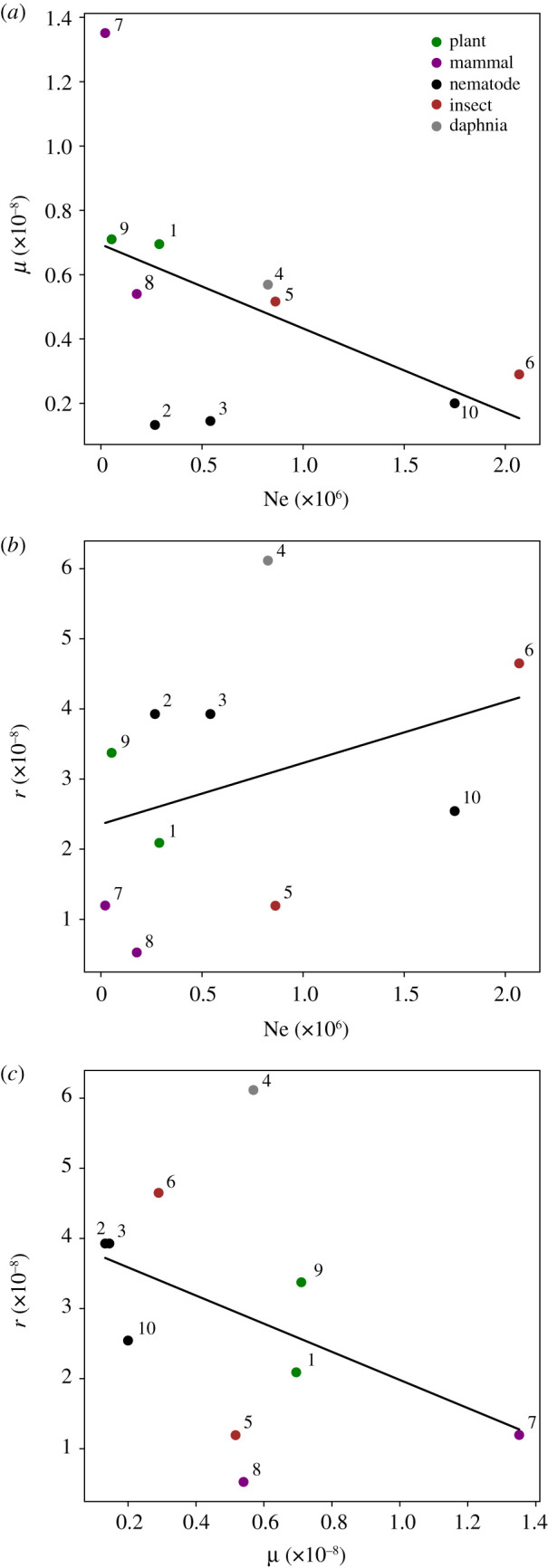
Relationships between mutation rates, recombination rates and effective population size in species where high-confidence estimates are available. Black lines represent simple linear regressions. Species include: 1. *Arabidopsis thaliana*, 2. *Caenorhabditis briggsae*, 3. *Caenorhabditis elegans*, 4. *Daphnia pulex*, 5. *Drosophila melanogaster,* 6. *Heliconius melpomene*, 7. *Homo sapiens*, 8. *Mus musculus*, 9. *Oryza sativa*, 10. *Pristionchus pacificus*. Effective population sizes and mutation rates were taken from Lynch *et al.* [53]; human and mouse recombination rates were calculated from the genetic maps in Bhérer *et al*. [54] and Cox *et al*. [55], respectively. All other recombination rates were taken from Stapley *et al*. [37]. (Online version in colour.)

One lineage with characteristics that might conform to the requirements of our model is humans. As in other mammals, genome-wide mutation rates in humans are high, genome-wide recombination rates are low and population sizes are low (figure 5). Furthermore, male recombination rates in humans are almost half those of females [56], male mutation rates are up to 20-fold higher [35] and mating systems in humans are thought to have been historically polygynous [52], contributing to a further potential reduction in the effective size of the sex-linked region. If these characteristics were shared by our mammalian ancestors, the neutral processes that we have modelled could perhaps have played a role in the loss of recombination on human or other mammalian sex chromosomes early on in their evolution. Of course, mammalian sex chromosomes are old and their various Y chromosome strata ceased to recombine hundreds of millions of years ago, so current patterns are not relevant to a process that might have been important in the initial expansion of a NRR. Bengtsson & Goodfellow [23] noted that recombination rates in the human PAR are particularly high and suggested that the maintenance of neutral sex-linked variation adjacent to the PAR boundary thus seemed unlikely. Given that at least one crossover is typically required per chromosome arm in order to ensure proper segregation during meiosis, chromosomes for which recombination has been lost over most of their length will, by necessity, possess high recombination rates in the PAR, as is the case for most mammalian sex chromosomes [57]. Thus, if the expansion of a NRR began under the influence of processes modelled here, it seems likely that the process would eventually become arrested by the increasing recombination rates in the shrinking PAR.

While it is not clear from our simulations whether the neutral processes modelled here could ever be solely responsible for the observed recombination loss on sex chromosomes, it seems plausible that the process could act in concert with other causes of recombination suppression. We found that regions evolving the complete loss of recombination were always flanked by regions of high *F*_ST_ between males and females and a low (but non-zero) recombination probability (e.g. figure 4). Consequently, such regions would experience LD with the SD locus. It is well known that the ability of selection to maintain a SA polymorphism on a sex chromosome depends on strong LD between the SA and SD loci [58]. Thus, the reduction of the recombination rate through neutral sequence divergence in the region around an already sex-linked region could facilitate selection for further reduction of recombination if a SA locus, a meiotic driver or a deleterious recessive mutation arose in this region [10,12]. Conversely, both positive and negative selective interference caused by selection for such genes near to the boundary of a NRR would reduce effective population size in this region [59], thus increasing the potential for the processes modelled here to play a role. Under such circumstances, neutral and non-neutral processes might mutually facilitate one another, increasing the likelihood or rate of recombination suppression. Of course, even in such cases, there are also several processes that can counteract the progressive loss of recombination on sex chromosomes, whatever its mechanism. For example, sex chromosome turnovers would erase any divergence between gametologs [20,60], as can sex reversals [61].

In conclusion, our simulations indicate that, in principle, recombination can be lost on sex chromosomes as a result of a neutral process of homologous sequence divergence in LD with a SD locus. Theoretically, this process should also apply to any region of a genome that is strictly heterozygous, e.g. around mating-type loci [62], supergenes such as those on the social chromosomes of certain ant species [63,64] or loci governing dimorphisms such as distyly in plants [65]. However, further work will be necessary to determine how often this process operates in nature. Our simulations predict that the rate and pattern of recombination loss, as modelled here, should be strongly affected by population size. It would therefore be interesting to compare patterns of recombination loss on sex chromosomes to past demographic histories, perhaps comparing lineages with orthologous sex determiners but differing extents of recombination loss. Ultimately, directly testing for the mechanism of recombination loss on sex chromosomes will be challenging. However, we argue that if we are to consider adaptive explanations, they must be set against a neutral null model. A case in point here is the commonly observed pattern of evolutionary strata on sex chromosomes. While it is often proposed that strata result from selection for linkage between SD and SA loci (e.g. [66]), it is clear that neutral processes can, in principle, result in similar patterns of sequence variation between gametologs when there are non-uniform ancestral recombination landscapes.

## Data accessibility

The simulation program written and used in this study is implemented in C++11. Full details and instructions for installation and running of this model can be found on GitHub (https://github.com/mscharmann/sexrecevo), along with python scripts used to plot the outputs of the model.
